# Loading-related injuries of mechanically loaded broilers under field conditions

**DOI:** 10.1016/j.psj.2025.105297

**Published:** 2025-05-15

**Authors:** Julia Unterholzner, Elke Rauch, Alexandra Blaeske, Michael Erhard, Anne Werner, Paul Schmidt, Martin Gotthart, Helen Louton

**Affiliations:** a Chair of Animal Welfare, Ethology, Animal Hygiene and Animal Husbandry, Department of Veterinary Sciences, Faculty of Veterinary Medicine, LMU Munich, Veterinaerstr. 13/R, D-80539 Munich, Germany; b Paul Schmidt, Statistical Consulting for Science and Research, Große Seestraße 8, D-13086 Berlin, Germany; c Verlade GbR, Eder 1, D-84332 Hebertsfelden, Germany

**Keywords:** Poultry, Animal welfare, Animal health, Mechanical loading, Injury

## Abstract

This publication is part of a large study whose objective was to assess animal welfare during 32 mechanical loadings of broilers. We here focus on animal health aspects and the influences of circumstances during mechanical loading. Broilers in two husbandry systems (HS) (mean number of fattening days: HS 2: 41.3 days; HS 3: 40.1 days) were assessed on-farm for loading-related injuries such as fractures, hematomas, and abrasions before and after mechanical loading. The influence of conveyor belt speed (fast vs. slow), container type (GP container vs. SmartStack container), HS, fattening method (FM), season, and sex on loading-related injuries was analyzed. The two HS were grouped according to the specifications of a retail trade label into three FM (HS 2: Standard and Standard Premium, HS 3: Premium), which differed, among other aspects, in genotype, stocking density, dark period, and access to a veranda. Hematomas on the wing (6.55%) were the most common type of injury followed by hematomas on the wing tip (6.17%), abrasions on the body (4.92%), abrasions on the wing tip (4.25%), severe wing injuries (1.13%), and hematomas on the wing proximal to the wing tip (0.38%). A reduction in injuries was achieved by a slow belt speed, the use of a SmartStack container, and loadings during spring and summer. Loading broilers of HS 3 compared with those of HS 2 led to a significantly lower risk of severe wing injuries, total abrasions, and wing tip abrasions. Broilers of the Standard Premium (*P* = 0.038) and Premium FM (*P* = <0.001) had a significantly lower risk of severe wing injuries than those of the Standard FM, demonstrating that not only the genotype, which is one of the major differences between HS 2 and 3, influences the injury rate. Other differences in FM, such as a longer dark period, a lower stocking density at housing, more enrichment, and access to a veranda should be considered as influencing factors.

## Introduction

At the end of a fattening period, broilers are caught and loaded for transport to the slaughterhouse. The process of catching and loading, as well as the subsequent transport to the slaughterhouse, can be associated with injuries and deaths (Cockram et al., 2018; Jacobs et al., 2017a; Kittelsen et al., 2015) and thus is a critical process. In 2023, 631,476,222 broilers, corresponding to a weight of 1,086,085,100 tons were loaded and slaughtered in Germany (DESTATIS, 2022). The global trend towards rising meat consumption and thus increasing meat production has continued almost steadily over the last few years. In 2021, meat production increased for all common animal species such as cattle, pigs, and poultry (FAO, 2021). Poultry meat production has been the global leader for many years, with a worldwide production of approximately 133.90 million tons of poultry meat in 2020 (FAO, 2021).

For transport to the slaughterhouse, broilers must be caught and loaded into crates. Loading broilers can be done either manually by trained staff or mechanically by a loading machine. In Germany, manual loading of broilers is the predominantly used method of loading. In Scandinavian countries, in contrast, broilers are mainly loaded by machines (Wessel et al., 2022). Loading machines have been developed to improve both animal welfare and animal health by reducing injuries and animal losses. Furthermore, machines are economically more profitable than manual loading (Gocke, 2000) and improve the working conditions for the catchers, as they only have to operate a machine instead of lifting thousands of broilers (Delanglez et al., 2025; Kilman, 2003; Knierim & Gocke, 2003). Several studies have shown that the bruising of wings and legs, which is associated with pain, can be reduced by using loading machines (Delezie et al., 2006; Farsaie et al., 1983; Knierim & Gocke, 2003; Lacy & Czarick, 1998). Furthermore, mechanical loading could lead to standardization and a gentler approach. Dutra et al. (2021) summarized the study results of several other authors and concluded that mechanized loading should be preferred to manual loading. In some European countries and the U.S., loading machines are already in use, and various forms of loading machines exist. However, the most common form of broiler loading in Germany currently is manual catching by commercial catching staff (Knierim & Gocke, 2003; Langkabel et al., 2015), and only 5% of German broiler chickens are caught using automated catching methods (Wolff, 2020). Thus, there are only few scientific evaluations available on the effects of mechanical loading methods. Furthermore, most former studies have assessed loading machines in study settings that compared manual vs. mechanical loading (Kittelsen et al., 2018; Langkabel et al., 2015; Mönch et al., 2020; Nijdam et al., 2005). This is why we only assessed mechanical loadings, but with differing potential influencing factors on animal health. Moreover, most former studies have assessed animal health after mechanical loadings on carcasses at the slaughterhouse. This is why we assessed animal health on-farm immediately after loading to exclude injuries that happen during transport, lairage, or unloading.

In previous studies, the majority of injuries resulting from catching and transport were fractures, dislocations, and bruises of legs, wings, and breast (De Koning et al., 1987; Jacobs et al., 2017b; Kittelsen et al., 2018; Langkabel et al., 2015; Mönch et al., 2020; Nicol & Scott, 1990; Nijdam et al., 2005). A detailed consideration of the factors influencing the frequency of those injuries shows that the occurrence of fractures and dislocations as well as of hematomas appears to be very complex. Gocke (2000) concluded that the speed of the conveyor belts and the type of crates in which the broilers are loaded are the most important factors for loading-related injuries during mechanical loading. Gocke (2000) assessed loading-related injuries after loading broilers with a “Chicken Cat” loading machine with belt speeds of the conveyor belts ranging from 0.80 m/s to 1.60 m/s and found that an increasing belt speed had an impact on the number of injuries to the broilers, especially for hematomas on the wing. Knierim and Gocke (2003) concluded that the effect of different transport containers should be considered when looking at injuries. To our knowledge, studies that compared different container types for their influence on animal health after loading do not exist. For this reason, different container types were included in the present study. Former studies found that, among other factors, genotype and ambient temperature may influence the occurrence of bruising (Kettlewell & Turner, 1985; Nijdam et al., 2004; Wessel et al., 2022). Knowles and Broom (1990) also found a relationship between the incidence of bruising and the temperature of the day. In a study by Wessel et al. (2022), the sex of manually loaded broilers had a major influence on the occurrence of severe wing injuries (fractures, epiphysiolyses, and dislocations) and bruising. The percentages of broilers with injuries after loading vary greatly between earlier studies. The result is affected by the method of loading, the method of recording (e.g., the size and type of injury), the timepoint at which the broilers were observed (e.g., after transport or after slaughter), whether the injuries were assessed by a veterinarian directly or recorded by a camera system at the slaughterhouse, or whether the data were obtained from meat inspection or quality control information (Knowles & Broom, 1990). The percentage of broilers with one or more severe wing injuries (SWI) after mechanical loading ranges from 0.15% (Musilová et al., 2013) to 1.88% (Jacobs et al., 2017b). The percentage of broilers with one or more hematomas after mechanical loading ranges from 0.04% (Knierim & Gocke, 2003) to 7.80% (Nijdam et al., 2005).

In a previous study by Mönch et al. (2020), advantages of loading with a loading machine were shown in comparison with manual loading regarding the health of the animals. Wolff et al. (2019) observed that mechanically loaded animals showed less wing flapping (50.90%) than manually loaded animals (68.60%) and therefore fewer bruises. Nevertheless, optimizations of the process with the loading machine are necessary to reduce wing flapping. As a possible risk factor for the increased occurrence of hematomas, besides the starting and stopping of the conveyor belts and the conveyor belt speed, the crating of the animals from the last conveyor belt into the container was identified as a potential risk area for injuries (Wolff et al., 2019).

The present study investigated the influence of various parameters on the occurrence of injuries that occur during the mechanical loading and crating of broilers (loading-related injuries). The assessed parameters were the influence of rotation speed of the conveyor belts (1,800 vs. 2,000 turns per minute), type of container (GP container vs. SmartStack container), season, and husbandry system or associated fattening method. Additionally, we wanted to consider if the modified version of the CMC Apollo Generation 2 loading machine results in fewer loading-related injuries than the use of the previous version evaluated by Mönch et al. (2020).

## Animals, materials and methods

### Animals and farms

**Fig. 1:**
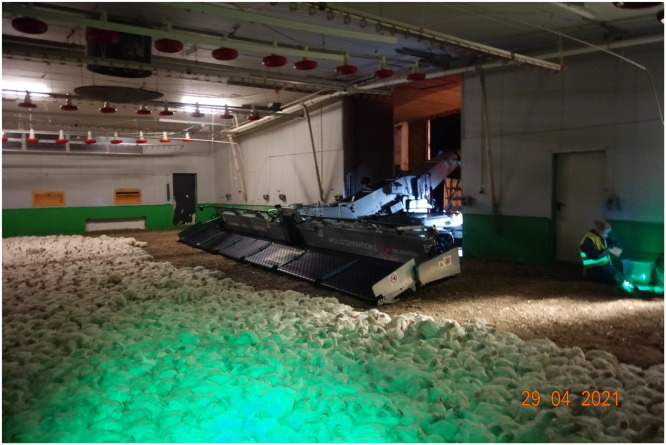
Modified version of the Apollo Generation 2 loading machine (CMC Industries-Ciemmecalabria, Cazzago S. Martino, Italy).

In this study, 32 mechanical loadings of broilers with a modified Apollo Generation 2 loading machine (CMC Industries-Ciemmecalabria, Cazzago S. Martino, Italy) (see Fig. 1) were assessed by trained veterinarians. This publication is part of a large study and presents the results on animal health of the assessed broilers. Another part of this study was published by Werner et al. (2023), who focused on animal behavior and used video recordings of the same flocks but not necessarily the exact same animals. Data collection took place from December 2020 to November 2021 on 10 broiler farms in Bavaria, Southern Germany. The flocks of the farms were of mixed sex (as hatched). All broilers were housed as day-old chicks and kept in accordance with the German Order on the Protection of Animals and the Keeping of Production Animals (2006). A detailed overview of the conditions on the farms and specifications of the 32 loadings is shown in Table 3 in Werner et al. (2023). Additional information on the farm conditions relevant for the health of the broilers in our study is presented in Table 1.

**Table 1:** Details of the 32 mechanical loadings (numbers of broilers assessed per container, cumulative mortality, number of thinning events) per husbandry system and fattening method.

HS^a^	FM^b^	Loading number	No. of broilers assessed per container	CM^c^ (%)	No. of thinning events
2	S	6	407	3.14	1
		13	469	2.14	1
		19	475	3.20	1
		21	478	2.14	1
		25	463	2.34	1
		27	465	2.66	2
		29	481	2.29	2
	SP	1	464	2.25	1
		2	428	3.64	1
		4	500	2.16	1
		10	530	1.63	1
		11	433	5.98	1
		16	415	1.93	1
		17	470	3.35	1
		18	518	3.54	1
		26	446	2.80	1
2 Ø^d^			465	2.82	1
	S Ø		463	2.56	1
	SP Ø		467	3.03	1
3	P	3	614	2.73	0
		5	608	1.36	1
		7	674	2.97	0
		8	586	1.30	0
		9	595	1.06	0
		12	641	1.39	0
		14	665	1.46	0
		15	691	2.65	0
		20	640	3.73	0
		22	688	1.81	0
		23	623	1.38	0
		24	591	2.23	0
		28	617	1.39	1
		30	669	1.24	0
		31	612	1.32	0
		32	618	1.77	0
3 Ø			633	1.86	0

Further details can be found in Werner et al. (2023) and Table 3 therein.

^a^HS, Husbandry system.

^b^FM, Fattening method; S, Standard; SP, Standard Premium; P, Premium.

^c^CM, Cumulative mortality.

^d^Ø, Average.

To use the collected data of a flock for the analysis, the following conditions had to be met: no medical treatment 10 days before loading, a cumulative mortality rate below 6%, and a stocking density below the individual upper limit for the fattening method (see Table 1 in Werner et al. (2023)). The barns had daylight and at least 3% window area in relation to the barn floor area. The light regime adhered to a minimum of 6 hours dark period (Standard fattening method) or a minimum of 8 hours dark period (Standard Premium and Premium fattening methods). During loading, the barn had to be darkened to a maximum of 1 lux to create uniform conditions. The farms fattened either the conventional fast-growing Ross 308 strain or a slow-growing genotype such as Ranger Classic (at one loading the Ranger Classic flock was mixed with Hubbard 787). Table 1 in Werner et al. (2023) gives an overview of the specifications and requirements (genotype, maximum stocking density, enrichment, veranda, feeding) of the husbandry systems and fattening methods and the number of participating farms. The husbandry systems were classified according to the specifications of the German retail trade’s “Haltungsformsiegel” (Haltungsform.de, 2023) into husbandry system 2 and husbandry system 3. In brief, husbandry system 2 housed fast-growing broilers, whereas husbandry system 3 housed slow-growing broilers. In 16 of the 32 loadings, i.e., 4 loadings per season, broilers that had been raised in husbandry system 2 conditions were assessed. In the other 16 loadings, broilers raised in husbandry system 3 were assessed. The classifications of the fattening methods followed the specifications of the Standard, Standard Premium, and Premium labels.

### Loading

The Apollo Generation 2 loading machine consists of 11 conveyor belts. The broilers are collected from the floor via the long and slightly inclined platform of the first six parallel conveyor belts (summarized as conveyor belt 1, about 9 m wide in total). From here, they get onto two conveyor belts (summarized as conveyor belt 2) that run perpendicular to conveyor belt 1 and transport the animals from the left and right sides to the center of the loading machine. A height difference of approximately 14 cm was measured between conveyor belt 1 and the slightly lower conveyor belt 2. The central part of the loading machine consists of three conveyor belts (summarized as conveyor belts 3 to 5) that transport the broilers to the rear end of the machine, where they are then loaded into individual crates. Hence, the broilers do not come into direct contact with the staff and remain in an upright position during the whole loading and crating process. The rear end of the machine is operated by two workers who can adjust its height and manually move it to the left and right to load the crates. The workers can set a target weight per crate for the machine, and the conveyor belts stop and a red-light signal is shown at the rear end of the central channel as soon as this target weight is reached. The target weight per crate is calculated by the slaughterhouse staff using the estimated weight of the broilers at transport time, which is given by the farmer 48 hours before the loading. The minimum area in square centimeters per kilogram live weight is given by the German Animal Welfare Transport Regulation (2009) (German designation: Tierschutztransportverordnung).

The containers are placed on a rotating platform on the back of the machine, which has space for up to three containers at a time and can be rotated mechanically to move containers. One or two forklift drivers, usually the farmers themselves, move the containers by a forklift truck, stack them on the transport vehicle, and bring empty containers to the rotating platform of the machine. The loading machine was moved slowly and carefully in a straight line towards the end of the barn via remote control by the worker standing in front of the machine supervising the collection of the broilers form the ground. Usually, it is necessary to drive the machine through the barn twice to be able to collect all animals because of the width of the barns. During the loading, the angle of inclination of the front of the machine is constantly checked by one or two workers. In addition, they make sure that the stocking density on the conveyor belts is appropriate, that the broilers can get from the floor onto the loading machine smoothly and that no dead broilers are being loaded. Therefore, for one mechanical loading, usually three workers operating the loading machine and one or two forklift drivers are needed. All of the workers involved in the loading process were officially certified by a veterinary office (includes agricultural education or schooling with exam by the veterinary office) according to article 17 of the German Order on the Protection of Animals and the Keeping of Production Animals (2006) (German designation: TierSchNutztV) or had comparable expertise. The entire staff operating the machine had thus been instructed, trained, or supervised by certified people.

Compared with the usual Apollo Generation 2 loading machine, the modified version of this loading machine has a modified horseshoe-shaped conveyor belt structure, which is less rough, less prominent, and possibly more slip resistant than the previous version, as well as easy to clean. In addition, the modified machine uses a “soft-go” mechanism with the intention to make the starting and stopping of conveyor belts gentler on the broilers. As the transition of the animals from the caging belt into the transport container represented a critical point and a crucial risk area for the occurrence of injuries in a previous study by Mönch et al. (2020), the modified loading machine has also been equipped with an improved module at the adjustable rear end of the machine, which makes it easier to adjust the angle of inclination.

Two rotation speeds and their influence on loading-related injuries were compared in our study. A speed of 1,800 turns per minute was defined as the slow rotation speed and 2,000 turns per minute as the fast rotation speed. The speed of the conveyor belts is influenced by the rotation speed that can be set manually on the machine. Furthermore, the speed of the conveyor belts increases continuously the closer the conveyor belt is located to the container platform on the back of the machine. This setting is necessary to ensure a smooth transition of animals from the front to the back.

At each loading, the broilers of one GP container and one SmartStack container were examined (usually: containers 12 and 14; loadings 1 to 3: containers 6 and 8). The order in which the container types were loaded was evenly alternated. The GP container (GP live bird container supply system, Marel, Gardabaer, Iceland) consists of a total of eight crates arranged in two parallel rows and four crate levels (Fig. 1 in Werner et al. (2023)). Hence, the two crates on one level are separated from each other by a central partition. This container can thus be loaded starting from the bottom, alternating sides, and one worker can carefully close the crate that has just been loaded while the other one continues loading. Each crate has a surface of 1.20 m². The average aimed weight of a loaded crate was 72.20 kg. Under conditions of practice on-farm, a crate is filled with 24 to 28 fast-growing broilers or 34 to 38 slow-growing broilers, depending on the mean weight of the loaded broilers, the ambient temperature, and transport conditions such as transport duration. The SmartStack container (SmartStack 5t1, Marel Poultry B.V., Boxmeer, Netherlands) (Fig. 1 in Werner et al. (2023)) consists of five crates arranged one above the other, and there is no central partition dividing the crates in half. Another difference to the GP container system is that the top crate of the SmartStack container is closed with a net instead of a pull-out plastic lid. Crates 1 to 4 of the SmartStack container have a surface of 2.92 m² each. To provide enough space for the forks of the forklift, the lowest crate has a surface of only 2.43 m² and is filled on average with 25 kg less. The average aimed weight of a loaded crate was 144.50 kg. In practice, a crate is filled with 48 to 56 fast-growing broilers or 68 to 76 slow-growing broilers, depending on the weight of the loaded broilers and the ambient temperature. For the bottom crate, the average aimed weight was 119.50 kg. This means that 39 to 45 fast-growing broilers or 56 to 63 slow-growing broilers were loaded in the bottom crate.

To identify a potential seasonal influence on the occurrence of loading-related injuries, the observations were evenly distributed over the four seasons (winter: December 1st to February 28th, spring: March 1st to May 31st, summer: June 1st to August 31st, fall: September 1st to November 30th). An overview of the classification of the four main factors (season, husbandry system, rotation speed, container type) can be found in Table 2 in Werner et al. (2023).

**Table 2:** Assessed variables before and after mechanical loading (modified according to Mönch et al., 2020).

Variable	Severity	Assessment method	Type of injury	Location on body	Type of variable	Explanation
Body weight	n/a	animal scale	n/a	n/a	continuous	assessment of 200 broilers before loading and of one fully filled GP container and one SmartStack container after loading
Sex	n/a	phenotypical characteristics	n/a	n/a	binomial	female/male
Injury	severe injury	visual assessment and palpation; pathologic examination after diagnosis via palpation	Fracture (including epiphysiolysis humeri or luxation)	wing (broilers with ≥1 SWI)	binomial	occurrence yes/no; on either wing, left or right
	minor injury	visual assessment	hematoma (≥0.5 cm in diameter)	wing (broilers with ≥1 HoWI; including HoWIT and HoWIpWIT)	binomial	occurrence yes/no; on either wing, left or right; any location on the wing
				wing tip (broilers with ≥1 HoWIT)	binomial	occurrence yes/no; on either wing, left or right
				wing proximal to wing tip (broilers with ≥1 HoWIpWIT)	binomial	occurrence yes/no; on either wing, left or right
	minor injury	visual assessment	abrasion (≥0.5 cm in diameter)	wing tip (broilers with ≥1 AoWIT)	binomial	occurrence yes/no; on either wing, left or right; any location on the wing
				body (broilers with ≥1 AoB)	binomial	occurrence yes/no; on the body

During assessment, the occurrence of severe and minor injuries was separately documented for the left and right wing; for analysis, data was used binomially for both sides.

Number of assessed broilers per loading: 200 before loading, on average 466 broilers of husbandry system 2 and 450 broilers of husbandry system 3 after loading; all broilers were randomly selected; broilers assessed before and after loading were not necessarily the same animals.

Abbreviations: SWI, severe wing injury; HoWI, hematoma on wing (including HoWIT and HoWIpWIT); HoWIT, hematoma on wing tip; HoWIpWIT, hematoma on wing proximal to wing tip; AoWIT, abrasion on wing tip; AoB, abrasion on body; n/a, not applicable.

Various precautions were taken before the loading. Within 72 hours before loading, an official veterinarian checked the broilers according to "Regulation (EC) No 854/2004 OF THE EUROPEAN PARLIAMENT AND OF THE COUNCIL" (2004). The feed was withdrawn by the farmers 2 to 4 hours before loading so that the crop would be empty, but the broilers would not be without feed for more than 12 hours at the time of slaughter. The broilers had access to water until right before the loading started. Two data loggers (LogBox RHT, B+B Thermo-Technik GmbH, Donauschlingen, Germany) were used to record the temperature inside and outside the barn. Up to 72 hours before loading, the farms deviated from the light regime and turned the light on continuously. Just before the barn was opened for the loading machine to enter, the workers dimmed the light and moved the broilers from the first meters of the barn by generating an air draft with a leaf blower to make the barn accessible for the machine. The workers then moved the machine into the barn and unfolded the machine arms. For the loading of the containers, the light intensity was set below 1 lux. This was measured with a Voltcraft LX-1108 luxmeter (Conrad Electronic, Hirschau, Germany).

### Assessments

Three veterinarians were trained, and two inter-observer reliability tests were performed on a total of 170 broilers after a loading in the preliminary phase of the study. The tests included a careful visual assessment and palpation of the broilers for severe or minor loading-related injuries on the wings, body, legs, and feet and the determination of the phenotypical sex of the broilers. Based on the collected data, the average percentage agreement of the examiners and the prevalence-adjusted bias-adjusted kappa (PABAK) value according to Byrt et al. (1993) were calculated to estimate the observer agreement. The PABAK value was calculated according to Gunnarsson (2000) with the formula: (k * p − 1) / (k − 1), where k = the number of categories, p = the relation between the observer agreements. The number of categories was always 2, as the categories were yes or no for each injury and male or female for the sex of each broiler. In addition, two complete test loadings including the pre-examinations in the barn had been performed before the actual data collection started to ensure a smooth procedure.

Within 24 hours before each loading, animal health in the flock was examined on-farm by the same three trained veterinarians. To ensure a random sampling and to calm the birds, the barn was dimmed to below 1 lux. To still be able to perform a thorough examination of the animals, the veterinarians used headlights. Two hundred broilers of each flock were examined for injuries and weighed using animal scales (Kern DE 35K5D, Kern & Sohn GmbH, Balingen, Germany or Mettler Toledo ICS425, Mettler Toledo GmbH, Giessen, Germany). The veterinarians also recorded the phenotypically apparent sex (head shape, thickness of feet, development and color of comb) of each broiler. In total, 6,400 animals were assessed as samples for the respective flock during the pre-examinations. During each pre-examination, 66 broilers were assessed in the front left of the barn, 68 broilers in the middle of the barn, and 66 broilers in the hind right of the barn to ensure an even distribution. In those areas, a random group of animals was separated for a short period for the examination to ensure that no broiler was assessed twice. The animals were carefully examined by visual assessment and palpated if necessary for minor and severe injuries as presented in Table 2. To exclude possible injuries unrelated to catching, we did not record bruises with greenish coloration. Greenish coloration of bruises indicates that the hematomas are older and in the process of healing and thus developed before catching (Hamdy et al., 1961). The body parts in which injuries of the broilers occurred were documented as follows: the wing tip as the area distal to the radial and ulnar ossa carpi, the wing as the entire wing except the wing tip, the trunk as the back and chest, the legs as the upper and lower leg, and the feet as the area distal to the tibiotarsus. For analysis, only the data of the variables shown in Table 2 was used.

During the loading of the fifth container of the barn, the belt speeds of the individual conveyor belts were measured three times each with a speed measurement device (Drehzahlmessgerät Testo 470, Testo SE & Co. KGaA, Lenzkirch, Germany). Conveyor belt 3 was not accessible to the speed measurement device. The same procedure was performed again during the loading of the fifteenth container.

Directly after being loaded, the broilers of two fully loaded containers (usually: containers 12 and 14 of a barn; loadings 1 to 3: containers 6 and 8) were examined by the veterinarians on-farm. The animals in both containers were assessed in a farm area well illuminated by spotlights according to the same criteria and by the same three trained veterinarians as in the pre-examination. Additionally, container type and crate number were recorded for each broiler. On farms with husbandry system 2, all broilers in both containers were examined, totaling 7,437 animals (GP container average: 211 broilers; SmartStack container average: 255 broilers). On farms with husbandry system 3, examination was limited to 25 broilers per crate in an old container and 50 broilers per crate in a new container owing to lack of time during the loading process. However, the remaining broilers in each crate were still weighed and evaluated by sex. In total, 7,175 broilers reared on farms with husbandry system 3 were examined after loading, on average 285 in a GP container and 348 in a SmartStack container.

Injuries were assessed manually and by palpation only, no x-rays were done. When broilers were diagnosed with severe injuries (fracture, dislocation), they were not transported to the slaughterhouse. To ensure animal welfare, those broilers were professionally stunned by concussion by trained staff and killed by cervical dislocation on-farm. The contralateral and uninjured wings were removed with secateurs and the humeri later assessed for their bone breaking strength. These results will be published separately.

The rejection rates of the respective loadings were provided by the slaughterhouse. Thus, an evaluation of rejection rates took place at loading level. A broiler was defined as rejected only if the entire broiler was classified as not suitable for consumption and the whole animal carcass was rejected.

To assess the study objective of the reduction in loading-related injuries by the modifications of the loading machine, the adjusted risks (AR) of injuries of 845 animals of the Standard fattening method, which were loaded into a GP container in winter, spring, and summer, were compared with those of the animals that were loaded by machine in the previous study by Mönch et al. (2020). The reduction of the dataset was necessary because in the previous study, no loadings had been assessed in fall, no containers of the SmartStack type had been used, and no animals of the Standard Premium or Premium fattening method had been examined. In addition, one test loading was performed with the common, unmodified version of the Apollo Generation 2 loading machine that had been used by Mönch et al. (2020). The reason was to assess animal health in the current heavy fattened broilers after being loaded with the former version of the machine.

### Statistical analysis

The focus of the analysis were the health of the animals during loading and the influences of selected parameters on the risk of loading-related injuries mentioned in Table 2. The loading-related injuries were categorized in SWI, hematomas on the wing (HoWI), hematomas on the wing proximal to the wing tip (HoWIpWIT), hematomas on the wing tip (HoWIT), abrasions on the body (AoB), and abrasions on the wing tip (AoWIT). The different injury outcomes were treated as binary variables and examined using mixed-effects logistic regression models. To account for potential farm-specific variability, farm membership was incorporated as a random intercept term. From these models, AR and corresponding 95% confidence intervals (CI) were estimated; risk comparisons were performed using risk ratios (RR). Potential predictors (namely, rotation speed, conveyor belt speed, container type, season, husbandry system, fattening method, sex, and rejection rate at the slaughterhouse) were alternately modeled as fixed effects. Continuous variables were standardized so that each RR could be interpreted as the change in risk associated with a one-standard-deviation increase in the variable. R software (version 4.1.2) was used to perform the statistical analyses.

## Results

### General data

The mean conveyor belt speed measured at the slow rotation speed settings was on average 1.02 m/s (0.93–1.20 m/s). The mean conveyor belt speed measured at the fast rotation speed settings was on average 1.20 m/s (1.10–1.31 m/s).

We examined 7,442 broilers of husbandry system 2, including 3,495 male, 3,943 female broilers, and 4 broilers that had a phenotypically non-assessable sex after loading. The male broilers weighed on average 2,922 g, the female broilers 2,558 g. For husbandry system 3, 7,175 broilers in total were examined after loading, including 3,550 male, 3,624 female broilers, and 1 broiler that had a phenotypically non-assessable sex. The male broilers weighed on average 2,215 g, the female broilers 1,853 g. The average weight of all broilers was highest in winter (2,501 g), followed by fall (2,394 g), spring (2,360 g), and summer (2,337 g). The outdoor temperature was on average 7.50°C (-9.00–27.70°C). The rejection rate at the slaughterhouse was on average 1.98% (0.35–10.29%).

### Inter-observer reliability test

For the first inter-observer reliability test, the PABAK value for SWI was 1.00, for HoWI 0.96, for HoWIT 0.97, and for the sex of the broilers 0.86. For the second inter-observer reliability test, the PABAK value for SWI was 1, for HoWI 1, for HoWIT 0.99, for AoB 0.97, and for the sex of the broilers 0.94. The training of the veterinarians before examination of the animals in the presented study was sufficient, and all values of the inter-observer reliability test were between 0.86 and 1.00.

### Pre-examination

During the pre-examination, a total of 6,400 animals were assessed. The order of observed injuries during the pre-examination with descending frequency was AoB (*n* = 17 broilers, AR: 0.26%, CI: [0.15; 0.45]), HoWI (n = 10 broilers, AR: 0.15%, CI: [0.07; 0.35]), HoWIT (n = 9 broilers, AR: 0.14%, CI: [0.06; 0.31]), AoWIT (*n* = 4 broilers, AR: 0.05%, CI: [0.01; 0.23]), HoWIpWIT (*n* = 1 broiler, AR: 0.00%), and no broiler had a SWI before loading. Thus, we can conclude that the injuries that were observed during the on-farm examination after loading happened during the process of loading.

### Loading-related injuries

Of the 14,612 examined broilers, 159 broilers (AR: 1.13%) had one or more SWI after loading, including 133 broilers of husbandry system 2 (AR: 1.33%) and 26 broilers of husbandry system 3 (AR: 0.27%) (Table 3). The SWI consisted of 90.60% epiphysiolyses humeri, 4.40% epiphysiolyses carporadioulnares, 3.10% other wing fractures, and 1.9% dislocations of the humeri. Furthermore, 819 broilers had one or more HoWI (AR: 6.55%), 55 had one or more HoWIpWIT (AR: 0.38%), and 771 had one or more HoWIT (AR: 6.17%) after loading. In addition, 631 broilers had an AoB (AR: 4.92%) and 526 broilers had an AoWIT (AR: 4.25%) after loading.

**Table 3:** Overview of the risks of loading-related injuries, subdivided into rotation speed, type of container, season, husbandry system, and sex, presented as percentage (%) and adjusted % accounting for farm-specific variation along with limits of the corresponding 95% confidence intervals (CI).

**Factor**	**Level**	**SWI**	**HoWI**	**HoWIpWIT**	**HoWIT**	**AoB**	**AoWIT**
		AwI/AO % 95% CI Adjusted % 95% CI	AwI/AO % 95% CI Adjusted % 95% CI	AwI/AO % 95% CI Adjusted % 95% CI	AwI/AO % 95% CI Adjusted % 95% CI	AwI/AO % 95% CI Adjusted % 95% CI	AwI/AO % 95% CI Adjusted % 95% CI
**Rotation speed (turns per minute)**	1,800	63/7,268 0.87 [0.67; 1.11] 0.94 [0.56; 1.59]	404/7,268 5.56 [5.04; 6.11] 6.83 [5.09; 9.11]	24/7,268 0.33 [0.21; 0.49] 0.33 [0.22; 0.49]	384/7,268 5.28 [4.78; 5.82] 6.59 [4.82; 8.94]	319/7,268 4.39 [3.93; 4.89] 5.07 [3.93; 6.52]	261/7,268 3.59 [3.18; 4.04] 4.23 [3.16; 5.64]
	2,000	96/7,344 1.31 [1.06; 1.59] 1.29 [0.79; 2.11]	415/7,344 5.65 [5.13; 6.20] 6.34 [4.75; 8.43]	31/7,344 0.42 [0.29; 0.60] 0.42 [0.30; 0.60]	387/7,344 5.27 [4.77; 5.81] 5.87 [4.31; 7.94]	312/7,344 4.25 [3.80; 4.73] 4.81 [3.75; 6.14]	265/7,344 3.61 [3.19; 4.06] 4.26 [3.21; 5.64]
**Type of container**	GP container	77/6,558 1.17 [0.93; 1.47] 1.18 [0.72; 1.96]	381/6,558 5.81 [5.26; 6.40] 6.88 [5.18; 9.07]	24/6,558 0.37 [0.23; 0.54] 0.37 [0.25; 0.55]	360/6,558 5.49 [4.95; 6.07] 6.52 [4.84; 8.72]	295/6,558 4.50 [4.01; 5.03] 5.20 [4.07; 6.62]	247/6,558 3.77 [3.32; 4.26] 4.47 [3.38; 5.90]
	SmartStack container	82/8,054 1.02 [0.81; 1.26] 1.11 [0.68; 1.83]	438/8,054 5.44 [4.95; 5.96] 6.69 [5.06; 8.80]	31/8,054 0.38 [0.26; 0.55] 0.38 [0.27; 0.55]	411/8,054 5.10 [4.63; 5.61] 6.29 [4.69; 8.40]	336/8,054 4.17 [3.75; 4.63] 4.84 [3.79; 6.15]	279/8,054 3.46 [3.08; 3.89] 4.16 [3.14; 5.47]
**Season**	Spring	27/3,644 0.74 [0.49; 1.08] 0.83 [0.43; 1.58]	194/3,644 5.32 [4.62; 6.10] 5.88 [4.56; 7.54]	16/3,644 0.44 [0.25; 0.71] 0.44 [0.27; 0.72]	181/3,644 4.97 [4.28; 5.72] 5.50 [4.22; 7.13]	143/3,644 3.92 [3.32; 4.61] 4.52 [3.39; 6.00]	116/3,644 3.18 [2.64; 3.81] 3.85 [2.79; 5.29]
	Summer	31/3,739 0.83 [0.56; 1.17] 0.97 [0.52; 1.79]	186/3,739 4.97 [4.30; 5.72] 5.80 [4.51; 7.43]	15/3,739 0.40 [0.22; 0.66] 0.40 [0.24; 0.66]	171/3,739 4.57 [3.93; 5.29] 5.34 [4.11; 6.92]	124/3,739 3.32 [2.77; 3.94] 3.91 [2.93; 5.19]	106/3,739 2.83 [2.33; 3.42] 3.41 [2.47; 4.68]
	Fall	48/3,633 1.32 [0.98; 1.75] 1.24 [0.68; 2.25]	304/3,633 8.37 [7.49; 9.32] 8.98 [7.15; 11.21]	12/3,633 0.33 [0.17; 0.58] 0.33 [0.19; 0.58]	294/3,633 8.09 [7.23; 9.03] 8.72 [6.88; 10.98]	178/3,633 4.90 [4.22; 5.65] 5.51 [4.22; 7.16]	161/3,633 4.43 [3.79; 5.15] 4.84 [3.60; 6.48]
	Winter	53/3,596 1.47 [1.11; 1.92] 1.75 [0.97; 3.13]	135/3,596 3.75 [3.16; 4.43] 4.32 [3.28; 5.67]	12/3,596 0.33 [0.17; 0.58] 0.33 [0.19; 0.59]	125/3,596 3.48 [2.90; 4.13] 3.95 [2.97; 5.25]	186/3,596 5.17 [4.47; 5.95] 5.91 [4.50; 7.71]	143/3,596 3.98 [3.36; 4.67] 4.79 [3.52; 6.48]
**Husbandry system**	2	133/7,437 1.79 [1.50; 2.12] 1.33 [1.01; 1.75]	488/7,437 6.56 [6.01; 7.15] 7.24 [5.50; 9.47]	28/7,437 0.38 [0.25; 0.54] 0.38 [0.26; 0.54]	464/7,437 6.24 [5.70; 6.81] 6.85 [5.12; 9.10]	420/7,437 5.65 [5.13; 6.20] 5.64 [4.69; 6.77]	354/7,437 4.76 [4.29; 5.27] 4.93 [3.97; 6.10]
	3	26/7,175 0.36 [0.24; 0.53] 0.27 [0.18; 0.43]	331/7,175 4.61 [4.14; 5.12] 4.47 [2.63; 7.51]	27/7,175 0.38 [0.25; 0.55] 0.38 [0.26; 0.55]	307/7,175 4.28 [3.82; 4.77] 4.16 [2.37; 7.22]	211/7,175 2.94 [2.56; 3.36] 3.00 [2.17; 4.13]	172/7,175 2.40 [2.06; 2.78] 2.41 [1.64; 3.53]
**Sex**	Male	45/7,044 0.64 [0.47; 0.85] 0.68 [0.40; 1.15]	281/7,044 3.99 [3.54; 4.47] 4.68 [3.50; 6.24]	15/7,044 0.21 [0.12; 0.35] 0.21 [0.13; 0.35]	267/7,044 3.79 [3.36; 4.26] 4.46 [3.28; 6.04]	255/7,044 3.62 [3.20; 4.08] 4.17 [3.24; 5.34]	203/7,044 2.88 [2.50; 3.30] 3.44 [2.58; 4.56]
	Female	114/7,565 1.51 [1.24; 1.81] 1.54 [0.95; 2.49]	537/7,565 7.10 [6.53; 7.70] 8.17 [6.23; 10.65]	40/7,565 0.53 [0.38; 0.72] 0.53 [0.39; 0.72]	503/7,565 6.65 [6.10; 7.23] 7.66 [5.75; 10.14]	376/7,565 4.97 [4.49; 5.48] 5.59 [4.41; 7.07]	323/7,565 4.27 [3.83; 4.75] 4.95 [3.78; 6.46]

Abbreviations: AwI, number of animals that showed the injury; AO, number of animals observed; SWI, severe wing injury; HoWI, hematoma on wing (including HoWIT and HoWIpWIT); HoWIT, hematoma on wing tip; HoWIpWIT, hematoma on wing proximal to wing tip; AoWIT, abrasion on wing tip; AoB, abrasion on body.

### Risks of loading-related injuries

An overview of the risks of loading-related injuries, subdivided into the analyzed potential factors of influence, i.e., rotation speed, type of container, season, husbandry system, and sex, is given in Table 3. A comparison of the factors influencing the occurrence of SWI, hematomas, and abrasions is shown in Table 4.

**Table 4:** Factors influencing the occurrence of severe wing injuries, hematomas, and abrasions, presented as estimated risk ratio (RR), limits of the corresponding 95% confidence interval (CI), and p-value.

Factor comparison	SWI	HoWI	HoWIpWIT	HoWIT	AoB	AoWIT
	RR 95% CI p-value	RR 95% CI p-value	RR 95% CI p-value	RR 95% CI p-value	RR 95% CI p-value	RR 95% CI p-value
**2,000vs. 1,800 turns per minute**	1.37 [0.93; 2.01] 0.112	0.93 [0.78; 1.10] 0.401	1.28 [0.75; 2.18] 0.366	0.89 [0.74; 1.07] 0.208	0.95 [0.79; 1.14] 0.563	1.01 [0.82; 1.23] 0.948
**SmartStack container vs. GP container**	0.94 [0.63; 1.39] 0.754	0.97 [0.83; 1.14] 0.730	1.05 [0.62; 1.79] 0.853	0.97 [0.82; 1.13] 0.668	0.93 [0.79; 1.10] 0.394	0.93 [0.77; 1.12] 0.435
**Spring vs. summer**	0.85 [0.39; 1.89] 0.957	1.01 [0.75; 1.37] 1	1.09 [0.44; 2.75] 0.994	1.03 [0.75; 1.41] 0.995	1.16 [0.82; 1.64] 0.707	1.13 [0.77; 1.66] 0.846
**Spring vs. fall**	0.67 [0.28; 1.61] 0.644	0.65 [0.49; 0.88] 0.002	1.33 [0.50; 3.54] 0.878	0.63 [0.46; 0.86] <0.001	0.82 [0.57; 1.18] 0.498	0.80 [0.53; 1.19] 0.472
**Spring vs. winter**	0.47 [0.23; 0.96] 0.032	1.36 [0.99; 1.86] 0.057	1.32 [0.49; 3.50] 0.889	1.39 [1.01; 1.93] 0.044	0.77 [0.56; 1.04] 0.109	0.80 [0.57; 1.14] 0.372
**Summer vs. fall**	0.78 [0.34; 1.81] 0.878	0.65 [0.48; 0.87] 0.001	1.21 [0.45; 3.28] 0.958	0.61 [0.45; 0.84] <0.001	0.71 [0.49; 1.03] 0.079	0.70 [0.47; 1.06] 0.121
**Summer vs. winter**	0.55 [0.29; 1.05] 0.084	1.34 [0.98; 1.83] 0.070	1.20 [0.45; 3.25] 0.964	1.35 [0.98; 1.87] 0.078	0.66 [0.48; 0.91] 0.004	0.71 [0.50; 1.01] 0.063
**Fall vs. winter**	0.71 [0.32; 1.56] 0.671	2.08 [1.50; 2.87] <0.001	0.99 [0.35; 2.82] 1	2.21 [1.58; 3.09] <0.001	0.93 [0.66; 1.31] 0.953	1.01 [0.69; 1.49] 1
**Husbandry system 2vs. husbandry system 3**	0.21 [0.13; 0.33] <0.001	0.62 (0.34; 1.11) 0.109	1.00 [0.59; 1.69] 0.999	0.61 [0.32; 1.14] 0.120	0.53 [0.37; 0.77] <0.001	0.49 [0.32; 0.75] 0.001
**Female vs. male**	2.26 [1.63; 3.13] <0.001	1.75 [1.52; 2.00] <0.001	2.48 [1.37; 4.49] 0.003	1.72 [1.49; 1.98] <0.001	1.34 [1.15; 1.57] <0.001	1.44 [1.21; 1.71] <0.001

Abbreviations: SWI, severe wing injury; HoWI, hematoma on wing (including HoWIT and HoWIpWIT); HoWIT, hematoma on wing tip; HoWIpWIT, hematoma on wing proximal to wing tip; AoWIT, abrasion on wing tip; AoB, abrasion on body.

**Fig. 2:**
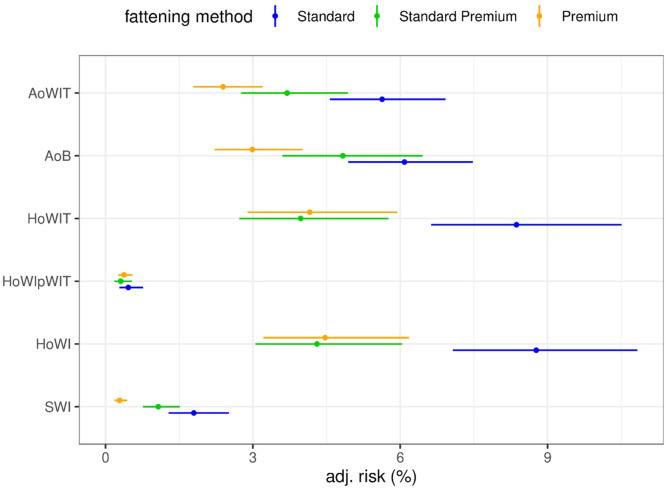
Effect of the fattening methods (Standard, Standard Premium, and Premium) on the risks of loading-related injuries in mechanically loaded broilers, presented as adjusted risk and the corresponding 95% confidence interval. Abbreviations: SWI, severe wing injury; HoWI, hematoma on wing (including HoWIT and HoWIpWIT); HoWIT, hematoma on wing tip; HoWIpWIT, hematoma on wing proximal to wing tip; AoWIT, abrasion on wing tip; AoB, abrasion on body.

**Fig. 3:**
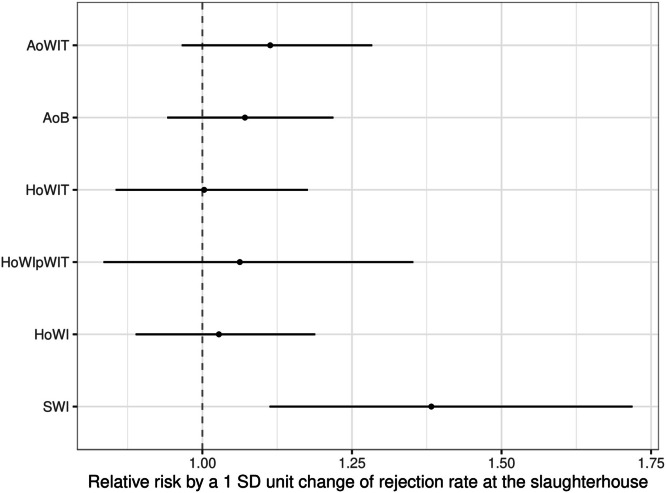
Effect of raising the factor rejection rate at the slaughterhouse by one SD unit on the risk of loading-related injuries in mechanically loaded broilers, presented as relative risk and the corresponding 95% confidence interval. Abbreviations: SWI, severe wing injury; HoWI, hematoma on wing (including HoWIT and HoWIpWIT); HoWIT, hematoma on wing tip; HoWIpWIT, hematoma on wing proximal to wing tip; AoWIT, abrasion on wing tip; AoB, abrasion on body.

The risk of SWI for broilers of husbandry system 2 increased when they were loaded at fast rotation speed compared with the slow rotation speed (1.72% vs. 1.03%; RR: 1.66, CI: [1.09; 2.53], *P* = 0.018). Increasing the mean conveyor belt speed by one SD unit (0.11 m/s) increased the risk of SWI in the Standard fattening method (RR: 1.50, CI: [1.11; 2.02], *P* = 0.008). For broilers of husbandry system 3, neither the rotation speed nor the mean measured conveyor belt speed influenced the risk of SWI. The container type (SmartStack container vs. GP container) did not influence the risk of SWI for broilers of any of the two assessed husbandry systems. Broilers of husbandry system 3 had a reduced risk of SWI compared with broilers of husbandry system 2 (0.27% vs. 1.33%; RR: 0.21, CI: [0.13; 0.33], *P* <0.001). Comparison of the fattening methods showed that broilers of the Standard Premium fattening method had a reduced risk of SWI compared with those of the Standard fatting method (1.07% vs. 1.80%; RR: 0.60, CI: [0.36; 0.98], *P* = 0.038). For broilers of the Premium fattening method, the risk of SWI was reduced compared with those of the Standard (0.29% vs. 1.80%; RR: 0.16, CI: [0.09; 0.29], *P* <0.001) or Standard Premium fattening method (0.29% vs. 1.07%; RR: 0.27, CI: [0.15; 0.48], *P* <0.001) (Fig. 2). Furthermore, the risk of SWI for broilers of husbandry system 2 was lowest in spring (AR: 1.01%), followed by summer (AR: 1.28%) and fall (AR: 1.61%). The highest risk of SWI was observed in winter (AR: 2.38%), with a statistically significant difference between winter and spring (2.38% vs. 1.01%; RR: 2.36, CI: [1.03; 5.38], *P* = 0.039). For broilers of husbandry system 3, no statistically significant influence of the seasons on SWI was detected. Male broilers of husbandry system 2 had a lower risk of SWI than female broilers of the same husbandry system (0.65% vs. 1.97%; RR: 0.33, CI: [0.22; 0.50], *P* <0.001). For broilers of husbandry system 3, sex had no effect on the risk of SWI. An increase in the rejection rate at the slaughterhouse by one SD unit (1.87) was associated with a higher risk of SWI during the preceding loading (RR: 1.38, CI: [1.11; 1.72], *P* = 0.004) (Fig. 3). An increase in body weight by 1 kg was associated with a higher risk of SWI for female broilers of the Standard (RR: 2.65%, CI: [1.00; 7.00], *P* = 0.050) and Standard Premium fattening methods (RR: 2.83%, CI: [1.07; 7.46], *P* = 0.036). For broilers of husbandry system 2, the risk of SWI was lower on fattening day 40 than on fattening day 43 (0.78% vs. 3.07%; RR: 0.25, CI: [0.08; 0.80], *P =* 0.010). For broilers of husbandry system 3, the number of fattening days had no effect on the risk of SWI. Raising the cumulative mortality rate by one SD unit (1.02) had no effect on the risk of SWI.

Regarding hematomas, neither the rotation speed nor the mean conveyor belt speed influenced the risk of HoWI, HoWIpWIT, or HoWIT. Loading the broilers of husbandry system 3 in a SmartStack container instead of a GP container decreased the risk of HoWI (4.12% vs. 5.24%; RR: 0.79, CI: [0.64; 0.97], *P* = 0.025) and HoWIT (3.75% vs. 4.94%; RR: 0.76, CI: [0.60; 0.96], *P* = 0.020). This risk-reducing effect could neither be seen for HoWIpWIT nor for animals of husbandry system 2. Looking at the husbandry systems, there seems to be a strong tendency that the risk of hematomas is higher for broilers of husbandry system 2 than for those of husbandry system 3, but without statistical significance. Comparing the fattening methods, we found that broilers of the Premium fattening method had a lower risk of HoWI (4.47% vs. 8.77%; RR: 0.51, CI: [0.32; 0.81], *P* = 0.002) and HoWIT (4.16% vs. 8.37%; RR: 0.50, CI: [0.30; 0.83], *P* = 0.004) than broilers of the Standard fattening method. Additionally, broilers of the Standard Premium fattening method had a lower risk of HoWI (4.30% vs. 8.77%; RR: 0.49, CI: [0.30; 0.79], *P* = 0.001) and HoWIT (3.97% vs. 8.37%; RR: 0.47, CI: [0.28; 0.80], *P* = 0.002) than broilers of the Standard fattening method (Fig. 2). The risk of HoWI was significantly increased for broilers of husbandry system 2 in fall compared with any other season. Compared with winter, in which the least HoWI were observed, the difference was 6.17 percentage points (10.78% vs. 4.61%; RR: 2.34, CI: [1.39; 3.94], *P* <0.001), compared with spring it was 5.24 percentage points (10.78% vs. 5.54%; RR: 1.94, CI: [1.16; 3.25], *P* = 0.005), and compared with summer it was 4.75 percentage points (10.78% vs. 6.03%; RR: 1.79, CI: [1.10; 2.91], *P* = 0.011). Similarly, the incidence of HoWI was greatest in fall for broilers of husbandry type 3, but the difference to other seasons was significant only in comparison with winter (6.11% vs. 3.13%; RR: 1.95, CI: [1.27; 2.99], *P* <0.001). Significant influences of seasons on the risk of HoWIpWIT were not detected for broilers of both husbandry systems. The risk of HoWIT was significantly increased in fall for broilers of husbandry system 2 compared with every other season. The difference was 6.44 percentage points compared with winter (10.61% vs. 4.17%; RR: 2.54, CI: [1.49; 4.35], *P* <0.001), 5.35 percentage points compared with spring (10.61% vs. 5.26%; RR: 2.02, CI: [1.19; 3.41], *P* = 0.003), and 4.96 percentage points compared with summer (10.61% vs. 5.65%; RR: 1.88, CI: [1.14; 3.09], *P* = 0.006). An increased risk of HoWIT in fall was also found in broilers of husbandry system 3 when compared with winter (5.73% vs. 2.84%; RR: 2.01, CI: [1.27; 3.18], *P* <0.001) and summer (5.73% vs. 3.70%; RR: 1.55, CI: [1.01; 2.37], *P* = 0.042). Male broilers of husbandry system 2 had a lower risk of HoWI (5.57% vs. 8.64%; RR: 0.65, CI: [0.54; 0.77], *P* <0.001) and HoWIT (5.30% vs. 8.15%; RR: 0.65, CI: [0.54; 0.78], *P* <0.001) than female broilers of the same husbandry system. Male broilers of husbandry system 3 had a lower risk of HoWI (2.88% vs. 6.02%; RR: 0.48, CI: [0.38; 0.60], *P* <0.001), HoWIpWIT (0.20% vs. 0.55%; RR: 0.36, CI: [0.15; 0.84], *P* = 0.019), and HoWIT (2.73% vs. 5.56%; RR: 0.49, CI: [0.39; 0.62], *P* <0.001) than female broilers of the same husbandry system. There was no relation between the percentage of hematomas at loading and the subsequent rejection rate at the slaughterhouse (Fig. 3). An increase in body weight by 1 kg was associated with a higher risk of HoWI (RR: 2.22%, CI: [1.27; 3.87], *P* = 0.005) and HoWIT (RR: 2.51%, CI: [1.43; 4.41], *P* = 0.001) for female broilers of the Premium fattening method. For broilers of neither husbandry system did the number of fattening days have an influence on the risk of hematomas. Raising the cumulative mortality rate by one SD unit (1.02) had no effect on the risk of hematomas.

Regarding abrasions, neither the rotation speed nor the mean conveyor belt speed had an effect on the risk of abrasions for broilers of husbandry system 2. For broilers of husbandry system 3, the risk of AoWIT was higher when loading with the fast rotation speed than loading with the slow rotation speed (2.55% vs. 1.80%; RR: 1.42, CI: [1.02; 1.98], *P* = 0.039). The container type (SmartStack container vs. GP container) did not have an influence on the risk of abrasions for broilers of any of the two assessed husbandry systems. Broilers of husbandry system 3 had a lower risk of AoB (3.00% vs. 5.64%; RR: 0.53, CI: [0.37; 0.77], *P* <0.001) and AoWIT (2.41% vs. 4.93%; RR: 0.49, CI: [0.32; 0.75], *P* = 0.001) than broilers of husbandry system 2. Comparing the fatting methods, we found that broilers of the Premium fatting method showed less abrasions than broilers of the Standard Premium and Standard fattening methods. Compared with broilers of the Standard fattening method, those of the Premium fattening method showed a lower risk of AoB (2.99% vs. 6.09%; RR: 0.49, CI: [0.32; 0.75], *P* <0.001) and AoWIT (2.39% vs. 5.63%; RR: 0.42, CI: [0.28; 0.65], *P* <0.001) (Fig. 2). The seasonal influence on the risk of abrasions was similar for loaded broilers of both husbandry systems, with the winter season showing the highest risks in comparison with the other seasons. For broilers of husbandry system 2, the risk of AoB was higher in winter than in summer (6.55% vs. 4.21%; RR: 1.55, CI: [1.05; 2.31], *P* = 0.021). For broilers of husbandry system 3, the risk of AoB was higher in winter than in spring (3.86% vs. 1.85%; RR: 2.09, CI: [1.17; 3.73], *P* = 0.006). The risk of AoWIT for these broilers was also higher in winter than in spring (3.13% vs. 1.36%; RR: 2.31, CI: [1.22; 4.36], *P* = 0.004). Male broilers of husbandry system 2 had a lower risk of AoB (4.85% vs. 6.32%; RR: 0.77, CI: [0.63; 0.93], *P* = 0.006) and AoWIT (4.02% vs. 5.70%; RR: 0.71, CI: [0.57; 0.87], *P* = 0.001) than female broilers of the same husbandry system. Male broilers of husbandry system 3 had a lower risk of AoB (2.47% vs. 3.51%; RR: 0.70, CI: [0.54; 0.92], *P* = 0.010) and AoWIT (1.92% vs. 2.88%; RR: 0.67, CI: [0.50; 0.90], *P* = 0.008) than female broilers of the same husbandry system as well. An increase in the rejection rate at the slaughterhouse by one SD unit (1.87) tended to be associated with a higher risk of AoB (*P* = 0.298) and AoWIT (*P* = 0.139) during the preceding mechanical loading, albeit without statistical significance (Fig. 3). An increase in body weight by 1 kg was associated with an increased risk of AoB (RR: 3.79%, CI: [2.07; 6.94], *P* <0.001) and AoWIT (RR: 4.16%, CI: [2.11; 8.23], *P* <0.001) for female broilers of the Premium fattening method. For broilers of husbandry system 2, the risk of AoB was lower on fattening day 41 than on fattening days 40 (4.01% vs. 6.37%; RR: 0.63, CI: [0.41; 0.97], *P* = 0.028) and 42 (4.01% vs. 6.54%; RR: 0.61, CI: [0.41; 0.92], *P* = 0.010). The risk of AoWIT was lower on fattening day 41 than on fattening day 42 for broilers of husbandry system 2 (3.59% vs. 5.68%; RR: 0.63, CI: [0.40; 1.00], *P* = 0.046). For broilers of husbandry system 3, the number of fattening days had no effect on the risk of abrasions. Raising the cumulative mortality rate by one SD unit (1.02) had no effect on the risk of abrasions.

## Discussion

### Loading-related injuries

One result of the present study is that 1.33% of the broilers of husbandry system 2 showed one or more SWI after loading. This is a relatively high percentage compared with the findings of previous studies, in which SWI occurred in 0.15–1.36% of broilers. Whereas Mönch et al. (2020) found 1.36% of broilers with one or more SWI after mechanical loading, Knierim and Gocke (2003) and Musilová et al. (2013), for example, reported much lower rates of wing fractures (on average 0.66% and 0.15%, respectively) in mechanically loaded broilers. This large span of results on SWI after mechanical loading indicates that multiple environmental and flock-specific factors (such as body weight, age, and health status of the broilers or daytime at loading) can influence the occurrence of injuries (Mönch et al., 2020). Most of the other studies assessed the broilers at the slaughterhouse and thus did not exclude injuries that happened during transport, lairage, or unloading. Also, the comparability with most studies is limited because most previous ones used a different type of loading machine or assessed animal health after manual loading and not mechanical loading.

Furthermore, the average weight of loaded animals is higher today than in previous studies. During the study by Mönch et al. (2020) (data acquisition: December 2016 to June 2017), the female broilers weighed 2,339 g and the male ones 2,726 g (all Ross 308). In the present study (data acquisition: December 2020 to November 2021), the female Ross 308 broilers weighed 2,588 g and the male ones 2,922 g, which is an increase in the average weight in fast-growing broilers by more than 200 g or 9% within 4 years. The results of former studies allow the conclusion that a high body weight contributes to a higher risk of SWI. Both Bingham (1986) and Langkabel et al. (2015) found that heavy broilers showed more lesions on the wing than those of the light weight class. We expected the percentage of broilers with one or more SWI to increase significantly in comparison with the study by Mönch et al. (2020), owing to the faster weight gain and higher average slaughter weight in the current study than in the study by Mönch et al. (2020). However, the percentage of broilers with one or more SWI is nearly unchanged (1.33% vs. 1.36%), indicating that the modifications of the loading machine that had been implemented before our study clearly improved the loading process. Also, in the current study, we found a strong influence of the weight of the broilers on loading-related injuries.

Our assessment of hematomas revealed that 6.55% of broilers showed one or more HoWI after loading. This is a relatively high percentage compared with other studies. The rate of hematomas after mechanical loadings in former studies ranged from 0.04% to 7.19%. Knierim and Gocke (2003) found that 0.04% of broilers had one or more hematomas after mechanical loading. However, bruises were only recorded if they had a minimum diameter of 2 cm, and the authors assessed the bruises in the slaughterhouse and not on-farm after loading. The mean corrected percentage of bruises in a study by Nijdam et al. (2004) was 2.20% after mechanical loading. Delezie et al. (2006) and Mönch et al. (2020), who compared manual with mechanical loading, found 4.20% and 7.19% of broilers, respectively, with one or more HoWI after mechanical loading. In the present study, hematomas were counted as such if the diameter was above 0.50 cm. This is very small compared with the size thresholds in other studies (Knierim & Gocke, 2003; Nijdam et al., 2005) and might explain the higher overall occurrence of hematomas. Most of the mentioned studies investigated the injuries at the slaughterhouse, as far as indicated by the authors. Thus, it is difficult to differentiate between injuries that resulted from loading and those that resulted from transport or handling at the slaughterhouse (Cockram et al., 2018). Additionally, the present study investigated feathered wings of live broilers instead of scaled carcasses. Comparability with results from other studies is furthermore limited because the previous studies did not use the same type of loading machine. Looking at the group (Standard fattening method, GP container type, and seasons winter, spring, and summer) comparable with the previous study by Mönch et al. (2020), it turned out that 5.54% of broilers had one or more HoWI. Of the mechanically loaded broilers in the study by Mönch et al. (2020) with the same loading machine without modifications, 7.19% of broilers showed one or more HoWI. Thus, in the present study, the risk of HoWI could be reduced in comparison with the previous study by Mönch et al. (2020) if the group with identical factors is compared. This finding emphasizes the positive effect of the modifications of the current version of the loading machine (soft-go method, modified loading module, and new conveyor belt structure) on the health of the broilers during loading.

### Risks of loading-related injuries

Our study focused on assessing risk factors during mechanical loadings only, whereas most former ones took place in a study setting in which manual und mechanical loading were compared. We found that significantly more broilers of husbandry system 2 had one or more SWI in loadings at fast than in loadings at slow rotation speed. Werner et al. (2023), who assessed the behavior of the broilers of the present study, found that wing flapping occurred more frequently with the fast than with the slow rotation speed and that the risk for a broiler to show wing flapping on a stopping or staring conveyor belt was 22 percentage points higher at fast than at slow rotation speed. Thus, not only the fast speed itself but also the harsher braking and accelerating of the conveyor belts at fast rotation speed led to an increased risk of wing flapping. Increased wing flapping on the loading machine can lead to a higher occurrence of loading-related injuries such as SWI. The reduced risk of loading-related injuries at a slower loading speed was also seen by Knierim and Gocke (2003), with 0.72% of the broilers showing one or more HoWI at a conveyor belt speed of 0.80 m/s compared with 1.33% at a belt speed of 1.6 m/s.

Wessel et al. (2022) found that wing flapping during loading increased the risk of hematomas. Furthermore, Werner et al. (2023), who assessed the behavior of the broilers of the present study during mechanical loading, found that wing flapping, bumping into an animal, and bumping against the machine or container increased the risk of hematomas and abrasions. In contrast to the effect on SWI, neither the rotation speed nor the conveyor belt speed had a significant influence on hematomas of the animals in our study. This result is surprising because, as already mentioned, Werner et al. (2023) found not only that wing flapping occurred more frequently but also that the risk for a broiler to show wing flapping on a braking or accelerating conveyor belt was significantly higher at fast than at slow rotation speed. Mönch et al. (2020) even found a smaller number of animals with one or more wing hematomas when increasing the rotation speed. For broilers of husbandry system 3 in our study, the risk of AoWIT was significantly higher at fast than at slow rotation speed. An explanation would be that the animals are brought out of balance more quickly at a fast rotation speed and then hit the conveyor belt while showing startling reflexes that can cause AoWIT. Overall, avoiding frequent stops and starts to smoothen the loading process (for example, with the soft-go method) appears to be more crucial to prevent minor wing injuries during mechanical loading than choosing the slow rotation speed.

Regarding the container system, the type of container used did not have an influence on the risk of SWI in the present study. However, fewer broilers of husbandry system 3 had one or more HoWI and HoWIT after loading into the SmartStack container than after loading into the GP container. The associated loading characteristics must also be considered when discussing the risks of loading-related injuries associated with both container types. Compared with the GP container, only four instead of seven pauses are required when loading the SmartStack container because of the smaller number of crates. However, loading takes overall longer owing to the higher stocking density per crate. The fewer braking and accelerating events could explain the reduced risk of hematomas for broilers in the SmartStack container. Moreover, compared with the GP container, the SmartStack container holds more broilers per crate, and only five instead of eight crates have to be closed. Knierim and Gocke (2003) found that opening and closing of the crates poses a further increased risk of injury and thus concluded that it is beneficial when opening and closing is limited. This factor could have contributed to the reduced occurrence of hematomas in broilers that were loaded into the SmartStack containers in our study. Wessel et al. (2022) found that avoiding striking the broilers against the containers during manual loading reduces the risk of wing flapping. Instead of eight individual crates (GP container), the SmartStack container consists of only five crates without a central partition. For this reason, the statistical probability of bumping against the container is lower, which would reduce the risk of wing flapping and thus the injury risk. Werner et al. (2023), who studied the same flocks we studied, found that the risk of wing flapping was almost the same with both container types but confirmed that the risk of bumping against the machine or the container was reduced when loading SmartStack containers compared with GP containers. Therefore, our results suggest that using SmartStack containers with no medial partition allowed us to reduce the risk of bumping into the container and thus lower the injury risk.

Our comparison of two husbandry systems revealed that 1.33% and 0.27% of the broilers of husbandry systems 2 and 3, respectively, showed one or more SWI. This significant difference in the risk of SWI might be explained by the frequent occurrence of abnormal skeletal development and the high rates of daily weight gain in fast-growing broiler hybrids (Bradshaw et al., 2002; Olkowski et al., 2011; Wideman & Prisby, 2013). Possibly, abnormal development of the epiphyseal plate caused by fast growth could result in reduced humeral stability. Such instabilities would be without consequences owing to low mechanical stress during rearing, until the process of loading. The higher weight of the broilers in husbandry system 2 (compared with those in husbandry system 3) likely contributed to mechanical stress. Moreover, other researchers have found that pre-existing pathological alterations, caused for example by mechanical damage to the growth plate of the humerus, can promote local bacterial colonization (Bradshaw et al., 2002; Dinev, 2012; Prisby et al., 2014; Wideman & Prisby, 2013). Such bacterial colonization implies additional weakening of the tissue in the growth plate. In addition to those findings, Werner et al. (2023), who studied the same flocks we did, found that broilers of husbandry system 3 showed a lower risk of bumping into an animal or against the machine or container than broilers of husbandry system 2. Fast-growing animals (as used in husbandry system 2) show less active behavior than slow-growing animals (Bokkers & Koene, 2003), and the inactivity of the animals increases with increasing weight (Dawson et al., 2021). In this respect, it is possible that the broilers in husbandry system 2 had fewer possibilities to react to external influences and thus to avoid a collision because of their higher weight. However, the influence of rearing conditions other than daily weight gain and genotype should not be neglected because a combination of various parameters, such as light program, barn stocking density, and access to a veranda, can significantly lower the risk of SWI during loading. Broilers of the Standard Premium fattening method in comparison with the Standard fattening method had a significantly lower risk of SWI in our study. Both fattening methods exclusively fattened the fast-growing Ross 308 hybrid, wherefore the genotype or daily weight gain is not the only decisive factor, but management factors also play a crucial role. However, Forseth et al. (2023) found more SWI in slow-growing Hubbard JA787 broilers than in fast-growing Ross 308 broilers (0.032% vs. 0.007%). The authors concluded that the higher activity level of slower growing broilers could even make them more prone to injuries and fractures. Also, they fattened the slow-growing broilers longer than the fast-growing broilers (46 vs. 33 days), which resulted in a higher mean weight (1.654 g vs. 1.357 g). The increased weight of the longer fattened slow-growing broilers, and therefore more mechanical load involved in any movement, may be associated with a higher risk of SWI. The fact that the slow-growing broilers in our study were fattened for as long as the fast-growing ones, and thus were lighter, explains the different results in comparison with our study. As we assessed loadings during regular farm practice, the genotype was necessarily linked to other farm-specific fattening conditions such as duration of the dark period, access to a veranda, number of enrichment materials, etc. The Standard Premium and Premium fattening methods differ in more aspects than just the genotype, which means that risk factors cannot be attributed to genetics alone. This is why we did not consider the genotype as a stand-alone factor.

Regarding the husbandry systems and fattening methods, we found an increased risk of hematomas and abrasions in broilers of the Standard fattening method. Kaiser and Smith (1958) found that some poultry breeds tend to bruise more easily than others and that about 37% of the variation in the percentage of hematomas was associated with differences in the degree of fleshing, the average weight of flocks, and the number of days the broilers were fattened. Their finding that poorly fleshed broilers tended to have fewer bruises than birds with better fleshing was confirmed by another study (Crothers Jr and Helbacka (1960) and aligns with our result that the fast-growing broilers of husbandry system 2 with more fleshing bruised more easily than the slow-growing broilers of husbandry system 3. Moreover, the explanation that the risk of injuries increased because animals of husbandry system 2 had fewer possibilities to react to external influences and thus to avoid a collision owing to their higher weight may account not only for SWI but also for the increased risk of hematomas and abrasions. Also, the environmental conditions during the fattening period seem to be crucial, as broilers of the Standard Premium fattening method had a lower risk of minor wing injuries than those of the Standard fattening method.

Winter and fall turned out to be the seasons with the highest rate of SWI in the present study. Additionally, the average weight of the broilers was highest in winter. There is a connection between heavier broilers and a higher risk of ascites, as ascites can be caused by insufficient oxygenation of the body due to the disproportionately small heart and lungs (Kalmar et al., 2013). This pre-existing condition could have led to a higher risk of injuries during loading. The higher percentage of heavier birds and more SWI in winter and the assumption of associated restricted blood circulation in the presented study thus aligns with the findings of both Olkowski et al. (2011) and Junghans et al. (2022), who found the highest rates of ascites during the colder months in their studies. Furthermore, Werner et al. (2023) found an increasing risk of wing flapping in fall in behavior observations of the flocks of the present study. An explanation can be that during the colder seasons the average weight of the loaded broilers is higher and leads to more physical restrictions (Bokkers & Koene, 2003) so that the broilers lose their balance more easily on the conveyor belts and react by flapping their wings (Werner et al., 2023).

Our seasonal analyses furthermore showed the highest risk of hematomas during fall. In the same flocks that we examined in the present study, Werner et al. (2023) found that wing flapping and bumping into an animal, which both increase the risk of hematomas, occurred most frequently in fall. We found the fewest hematomas in winter, which could be due to vasoconstriction especially in the tip of the wings when the broilers are exposed to cold temperatures. Hamdy et al. (1961) found that after trauma, bruising was less apparent at low ambient temperature and more apparent at high ambient temperature and that increased blood flow to the skin surface in warm conditions and restriction of blood flow to the surface to conserve heat in cold conditions can influence the severity of superficial hemorrhage after trauma. In addition, broilers caught at lower temperatures show a less active behavior, resulting in fewer physical injuries during catching and transport and a lower percentage of bruising (Hamdy et al., 1961; Nijdam et al., 2004).

In the present study, female broilers of husbandry system 2 loadings had a higher risk of SWI than male broilers. Even though faster growth and leg limb abnormalities were more often seen in male than in female broilers in former studies (Bradshaw et al., 2002; Classen & Riddell, 1989), several studies (Mönch et al., 2020; Wessel et al., 2022) also found that female broilers show more wing fractures after loading than male broilers. With the one-leg manual catching method, 77.40% of the broilers with epiphysiolysis and 60.70% of the broilers with one or more hematomas were female in the study by Wessel et al. (2022). Possibly, hormonal differences between female and male broilers cause differences in skeletal development, along with an increased risk of epiphyseal rupture in female broilers (Mönch et al., 2020). Müsse et al. (2022) found that female broilers had shorter and lighter tibiotarsi with a smaller minimum diameter and a lower breaking strength of the bones. The lower breaking strength of the bones would be an explanation for the frequent occurrence of epiphysiolysis in female broilers. Also, behavioral differences between male and female broilers could be one factor that contributes to a higher risk of SWI in female broilers, as studies have shown that female broilers tend to react more anxiously to stressful stimuli than male broilers (Nätt et al., 2014; Werner et al., 2023).

In the present study, female broilers of both husbandry systems had a significantly higher risk of minor wing injuries than male broilers of the same husbandry system. These findings align with those of Mayes (1980) and Nijdam et al. (2004) and may be explained by differences in bone stability (Müsse et al., 2022) and behavior (Nätt et al., 2014; Werner et al., 2023). Furthermore, Taylor and Helbacka (1968) found that female broilers had significantly higher breast skin and tissue bruising scores than the male ones. Also, behavioral differences between male and female birds influence the risk of bruising during mechanical loading. Werner (2024) found that female broilers showed more defensive stress-related behavior in pre-loading examinations of the same flocks we studied, especially during the avoidance distance test. A higher value in the avoidance distance test was found to be positively correlated with a higher risk of wing flapping during mechanical loading, which was positively correlated with a higher risk of minor wing injuries in our study.

In our study, the rejection rate at the slaughterhouse ranged from 0.35% to 10.29% and was on average 1.98%. This rate is close to the average percentage of 2.10% that is reported in Germany (DESTATIS, 2022). In comparison with other studies (Gerrits et al., 1985; Jespersen, 1982; Kettlewell & Turner, 1985), in which the range for the rejection rate was between 5% and 30%, the rejection rate in the current study appears to be quite low. We found a positive correlation between SWI and the rejection rate at the slaughterhouse. Both aspects are essential for animal welfare and important for the farmers from an economic point of view. In Germany, deep dermatitis is the main reason for rejection at the slaughterhouse, accounting for nearly half (0.63% of 1.48%) of the rejections on average (Junghans et al., 2022). Deep dermatitis is caused by bacterial infection, and pre-existing health conditions such as *Escherichia coli* septicemia, which is associated with deep dermatitis, could cause infections of the bone marrow and decrease the strength of the skeleton, thus raising the risk of SWI. Moreover, sick broilers might be less able to position themselves on the conveyor belts and keep their balance.

## Conclusion

In our study, the fast rotation speed of the conveyor belt led to increased occurrence of SWI in broilers of husbandry system 2 and increased numbers of broilers with one or more AoWIT in husbandry system 3, as compared with the slow rotation speed. Consequently, loading of broilers at a slow rotation speed can be recommended. A slow and soft acceleration and braking during mechanical loading is crucial. For animals of husbandry system 3, HoWI and HoWIT occurred less often in the SmartStack than in the GP container. Therefore, especially when loading the more active slow-growing breeds, container modules such as the SmartStack container should be preferred. Broilers of husbandry system 2 and especially of the Standard fattening method have a significantly high risk of loading-related injuries and should be loaded with extra care and diligence, and all additional factors that have been found to increase the risk of loading-related injuries should be minimized. The risk of SWI and hematomas is highest in winter or respectively fall for both husbandry systems. Further research is needed to assess the influence of the conditions during rearing, catching, and loading on loading-related injuries. With better knowledge about the influence of housing conditions and possibly pre-existing health conditions, the risk can hopefully be reduced and therefore animal welfare improved.

## Ethical statement

The work described in this article with research on live animals was conducted in accordance with the principles and specific guidelines presented by the Institutional Animal Care and Use Committee (IACUC). All animals examined in this study were housed under conditions that comply with all governmental requirements; the loadings examined were performed within standard procedures used in conventional housing and loading of broilers.

The study was approved by the ethics committee of the veterinary faculty of the Ludwig Maximilian University in Munich, Germany (protocol number: 217-08-06-2020). All procedures used were in accordance with the German Order on the Protection of Animals and the Keeping of Production Animals (2006).

## Disclosures

The authors wish to confirm that there are no known conflicts of interest associated with this publication. The authors declare that the research was conducted in the absence of any commercial or financial relationships that could be construed as a potential conflict of interest.
